# Identification of Down-Regulated ADH1C is Associated With Poor Prognosis in Colorectal Cancer Using Bioinformatics Analysis

**DOI:** 10.3389/fmolb.2022.791249

**Published:** 2022-03-01

**Authors:** Ming Li, Ziming Liu, Jia Song, Tian Wang, Hongjie Wang, Yanan Wang, Jiguang Guo

**Affiliations:** ^1^ School of Basic Medical Sciences, Hebei University, Baoding, China; ^2^ College of Clinical Medicine, Hebei University, Baoding, China; ^3^ Affiliated Hospital of Hebei University, Baoding, China; ^4^ Department of Pathology, Affiliated Hospital of Hebei University, Baoding, China

**Keywords:** ADH1C, bioinformatics analysis, gene expression Omnibus database, colorectal cancer, differentially expressed gene

## Abstract

Colorectal cancer (CRC) is the second most deadly cancer in the whole world, with the underlying mechanisms largely indistinct. Therefore, we aimed to identify significant pathways and genes involved in the initiation, formation and poor prognosis of CRC using bioinformatics methods. In this study, we compared gene expression profiles of CRC cases with those from normal colorectal tissues from three chip datasets (GSE33113, GSE23878 and GSE41328) to identify 105 differentially expressed genes (DEGs) that were common to the three datasets. Gene ontology and Kyoto Encyclopedia of Genes and Genomes pathway analyses showed that the highest proportion of up-regulated DEGs was involved in extracellular region and cytokine-cytokine receptor interaction pathways. Integral components of membrane and bile secretion pathways were identified as containing down-regulated DEGs. 13 hub DEGs were chosen and their expression were further validated by GEPIA. Only four DEGs (ADH1C, CLCA4, CXCL8 and GUCA2A) were associated with a significantly lower overall survival after the prognosis analysis. Lower ADH1C protein level and higher CXCL8 protein level were verified by immunohistochemical staining and western blot in clinical CRC and normal colorectal tissues. In conclusion, our study indicated that the extracellular tumor microenvironment and bile metabolism pathways play critical roles in the formation and progression of CRC. Furthermore, we confirmed ADH1C being down-regulated in CRC and reported ADH1C as a prognostic predictor for the first time.

## Introduction

Colorectal cancer (CRC) causes approximately 10% of cancer-related deaths each year and is the second most common lethal cancers globally (Dekker et al., 2019; Sung et al., 2021). During the past few decades, the incidence of CRC has increased between two- and four-fold in many Asian countries, including China (Nielsen et al., 2005). Early CRCs are highly treatable and screening for the disease can greatly reduce cancer mortality (Schoen et al., 2012). However, CRC is often diagnosed at the advanced stage due to the limitations of the current screening methods and the high metastatic potential of CRC (Li et al., 2018a). Therefore, it is extremely important to identify more reliable biomarkers for the early diagnosis of CRC and to reveal the underlying pathogenic mechanism of CRC.

In recent years, high-throughput sequencing technology is particularly powerful for screening differentially expressed genes (DEGs) in biological samples (Vogelstein et al., 2013). This has led to an increase in the number of gene expression profiles researches and a huge amount of data have been accumulated in public databases, of which bioinformatic analysis is necessary to obtain valuable insights into disease mechanisms. This is also the case with CRC (Isella et al., 2015) and a lot of data on CRC-related DEGs have been accumulating in the database. Bioinformatics studies on CRC, based on public databases (Guo et al., 2017), have shown that using these methods will help to explain the formation of the disease and reveal the underlying molecular mechanisms.

In this study, we downloaded three original expression microarray datasets: GSE33113, GSE23878, and GSE41328, from the Gene Expression Omnibus (GEO) database (Available online: https://www.ncbi.nlm.nih.gov/geo). These datasets provided 127 CRC cases and 32 normal colorectal tissue samples. To obtain the common DEGs from the three datasets, we used the GEO2R online tool, which is linked with the GEO database and the Draw Venn diagram online software. The Database for Annotation, Visualization, and Integrated Discovery (DAVID) software was then applied to the Kyoto Encyclopedia of Genes and Genomes (KEGG) pathways and gene ontology (GO) enrichment analysis of these common DEGs. The GO analysis included biological process (BP), cellular component (CC), and molecular function (MF) categories. We also built the protein-protein interaction (PPI) network for the common DEGs and determined the hub genes using Cytoscape plugin cytoHubba. The expression of these hub genes, compared between CRC and normal colorectal tissues, was validated using the Gene Expression Profiling Interactive Analysis (GEPIA) (*P* < 0.05). We imported the validated hub genes into the UALCAN and OncoLnc online resource to perform the prognostic analysis (*P* < 0.05). Four DEGs (ADH1C, CLCA4, CXCL8, and GUCA2A) were found to be associated with a significantly worse prognosis and lower overall survival. The down-regulated protein level of ADH1C (Class I Alcohol dehydrogenase 1C, gamma polypeptide) was verified in the CRC tissues and normal colorectal tissues by immunohistochemical staining and western blot. Therefore, we reported ADH1C as a prognostic predictor for CRC for the first time. In conclusion, our bioinformatics study provides some significant pathways and potential biomarkers that may be helpful in the interpretation of the molecular mechanism of CRC and could provide potential markers for CRC diagnosis.

## Material and Methods

### Information From Three Microarray Datasets

We downloaded three original expression microarray datasets: GSE33113, GSE23878, and GSE41328, from the GEO database (Available online: https://www.ncbi.nlm.nih.gov/geo). These datasets provided 127 CRC cases and 32 normal colorectal tissue samples. The microarray data were all based on GPL570 Platforms (HG-U133_Plus_2 Affymetrix Human Genome U133 Plus 2.0 Array). The GSE33113 dataset included 90 CRC tissues and six normal colorectal tissues. The GSE23878 dataset included 36 CRC tissues and 24 normal colorectal tissues. The GSE41328 dataset included two CRC tissues and two normal colorectal tissues.

### Identification of Common DEGs

The DEGs between CRC tissues and normal colorectal tissues were first processed through the GEO2R online tools (Davis and Meltzer, 2007), with an adjusted P-value < 0.05 and logFC < −2 or logFC >2 as the cut-off criterion. The integrated raw data stored in TXT files were analyzed by Draw Venn diagram online software (http://bioinformatics.psb.ugent.be/webtools/Venn/) to obtain the common DEGs from the three databases. The DEGs with a log FC < 0 were deemed to be down-regulated genes, while the DEGs with a log FC > 0 were deemed to be up-regulated genes.

### GO and KEGG Pathway Enrichment Analysis of the Common DEGs

GO analysis is a widely used approach for the identification of unique biological characteristics of genes and proteins from data obtained by high-throughput sequencing (Ashburner et al., 2000). The KEGG is a group of databases that are designed to systematically analyze gene functions and link genomic information with higher-order biological function pathways (Kanehisa and Goto, 2000). The DAVID software is an integrative online bioinformatics program that aims to analyze and interpret the biological properties of genes or proteins (Huang da et al., 2009). Therefore, the GO and KEGG pathways enrichment analyses of common DEGs were carried out using DAVID 6.8 (https://david.ncifcrf.gov/). A P value <0.05 was used as the cut-off criterion.

### Protein-Protein Interaction Network Analysis

The PPI network of common DEGs was evaluated using STRING (Search Tool for the Retrieval of Interacting Genes), which is an open online tool (Szklarczyk et al., 2015). The PPI network complex of the common DEGs was then imported into Cytoscape, which is a free software for visualization of PPI networks to detect the hub DEGs (confidence score ≥0.4, maximum number of interactors = 0) (Shannon et al., 2003). The Cytoscape application, cytoHubba, was applied to determine the hub genes of the PPI network and the top 15 hub genes was ranking by the Maximal Clique Centrality (MCC) algorithm (Wang et al., 2021).

### Validation and Survival Analysis of Hub Genes

The GEPIA website was chosen for validation of the expression level of hub genes that were compared between the CRC tissues and normal colorectal tissues. The GEPIA software was used to collect high-throughput sequencing data from many biological samples of The Cancer Genome Atlas (TCGA) and the Genotype-Tissue Expression (GTEx) projects (Tang et al., 2017). The UALCAN website provided easy access to available cancer OMICS data (TCGA and MET500) (Chandrashekar et al., 2017) and OncoLnc was used as an online tool for the exploration of survival correlations based on TCGA (Anaya, 2016). We could find the P-value on the plot. A P value of <0.05 was used as the cut-off criterion.

### Patients and Specimens

We collected 18 pairs of formalin-fixed paraffin-embedding CRC tissues and matched normal colorectal tissues (13 men and 5 women; age range 43–84 years; mean age ±standard deviation (SD), 67 ± 14 years) from the Affiliated Hospital of Hebei University, among which 8 patients (3 men and 5 women; age range 53–84 years; mean age ±SD, 70 ± 12 years) had fresh CRC and matched normal colorectal tissues frozen in liquid nitrogen within 30 min after resection. All the CRC patients involved in the study were diagnosed with pathological proof and has not been received chemotherapy or radiotherapy before the surgery. The study was authorized by the Ethics Committee of Affiliated Hospital of Hebei University (HDFY-LL-2021-013). Informed consent was obtained from the patients and their families who participated in the study.

### Immunohistochemical Staining

The formalin-fixed paraffin-embedding CRC tissues were fixed in 10% formalin, embedded in paraffin, and sliced into 5 μm thick sections. The slides were deparaffinized by xylene and discontinuous concentration of alcohol. After incubating the slices at 95∼100°C for 10 min to retrieve antigens, the samples were cooled to room temperature treated with 3% hydrogen peroxide to block the endogenous peroxidase. Then, the primary antibody of ADH1C (A8081, Abclonal, Wuhan, China, 1:100) and CXCL8 (A2541, Abclonal, Wuhan, China, 1:100) were added to the slices and incubated in a humidified chamber at 4 °C overnight. For the next, the slices were incubated with the HRP*Polyclonal Goat Anti-Rabbit IgG (H + L) (PV-9001, Origene, Beijing, China) at room temperature for 20 min, followed by treatment with DAB substrate solution (ZLI-9019, Origene, Beijing, China). Finally, the slices were incubated with hematoxylin to visualize cell nuclei, dehydrated by alcohol, cleared in xylene, and sealed by using a mounting solution (neutral resin). The assay ensured no signal in the negative control. The result was observed by microscopy and analyzed by ImageJ 1.8.0 and IHC Toolbox plugin (Shu et al., 2016). Briefly, a proper threshold was stetted according to the positive DAB-staining specimen, which will be applied to all the pictures for comparison. The pixels of the area above the threshold, which is considered the positive area were measured automatically, and the % relative area which is the percentage of positive area of the whole picture was used to represent the immunoreactivity of the stained antibody. For each sample at least ten fields were measured.

### Western Blot

After the evaporation of the liquid nitrogen, the frozen tissues were homogenized by the homogenizer (02642, PRO Scientific, CT, United States) in the lysis buffer (P0013b, Beyotime, Shanghai, China) with phenylmethanesulfonyl fluoride on ice. Then the suspensions were centrifuged at 4°C 14,000×g for 15 min. The concentration of the total protein was measured by the BCA protein quantification kit (PA101-01, Biomed, Beijing, China). SDS-PAGE electrophoresis was used to separate the equal amounts of protein samples, which were then transferred to a nitrocellulose membrane (HATF00010, Millipore, MA, United States). The nitrocellulose membrane was blocked by the 5% nonfat milk (LP0031B, Solarbio, Beijing, China) diluting in tris-buffered saline with Tween-20 (TBST) at room temperature for 1 h, and followed by incubating with the primary antibodies of ADH1C (A8081, Abclonal, Wuhan, China, 1:1000), CXCL8 (A2541, Abclonal, Wuhan, China, 1:1000), and β-actin (20536-1-AP, Proteintech, Wuhan, China, 1:3000) as the internal reference at 4°C overnight, which was also diluted in TBST. At last, the nitrocellulose membrane was incubated with the secondary antibody (SA00001-2, Proteintech, Wuhan, China, 1:5000) for 1 h at room temperature and visualized by the UItraECL Substrate chemiluminescence detection Kit (Biomed, Beijing, China). The absolute intensity of the blot signals was quantified using the ImageJ 1.8.0 software (National Institutes of Health, Boston, MA, United States).

### Quantitative Real-Time PCR

As soon as the liquid nitrogen evaporated, the frozen tissue samples were homogenized by the homogenizer (02642, PRO Scientific, CT, United States) and lysed by the Trizol (CW0580, CWbio, Beijing, China) according to the protocol of the manufacturer. 1 μg of total RNA was used for the reverse transcription using the RT reagent kit with gDNA Eraser (RR047A, Takara, Dalian, China). After that, UltraSYBR Mixture (CW0957M, CWbio, Beijing, China) was used to carried out real-time PCR on CFX96 Optics Module (C1000, Bio-Rad, Singapore). The results were analyzed by the Bio-Rad CFX Manager 3.1 software and calculated by 2-ΔΔCt equation. The internal gene is β-actin. The sequence of the primers that used in the real-time PCR were as following: (ADH1C primers: forward 5′-GGA​CGC​ACG​TGG​AAA​GGA​G-3′ and reverse 5′-GAG​CGA​AGC​AGG​TCA​AAT​CC-3′; CXCL8 primers: forward 5′-CCA​AAC​CTT​TCC​ACC​CCA​AA-3′ and reverse 5′-TTC​TGT​GTT​GGC​GCA​GTG​T-3′; β-actin primers: forward 5′-CAT​GTA​CGT​TGC​TAT​CCA​GGC-3′ and reverse 5′-CTC​CTT​AAT​GTC​ACG​CAC​GAT-3′, and synthesized by Sangon (Shanghai, China).

### Statistical Analysis

Paired t-test was used to compare the quantitative data of the IHC, real-time PCR and western blot that conformed to normal distribution by the Graphpad 6.0. The results were represented by mean ± SD. A P value of <0.05 was used as the cut-off criterion.

## Results

### Identification of Common DEGs in CRC

**FIGURE 1:**
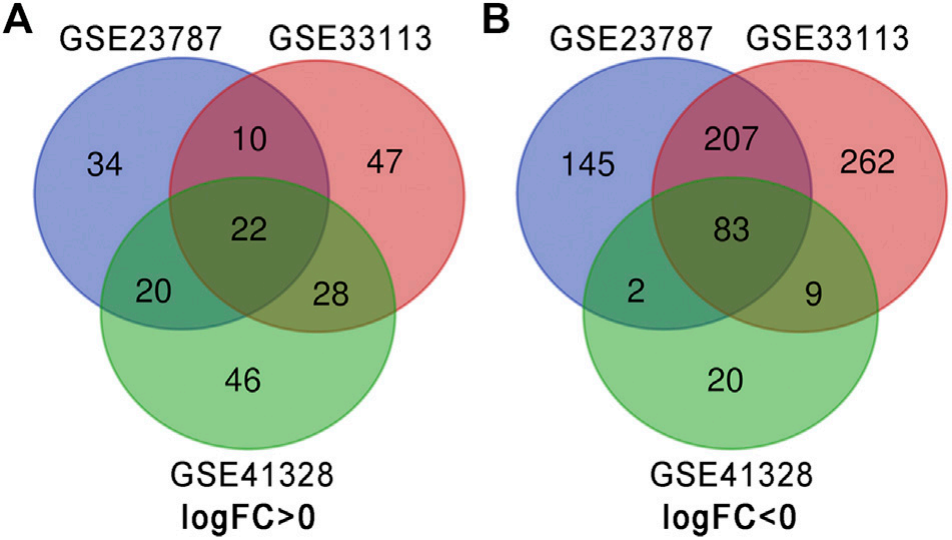
Identification of DEGs in the three datasets using the Draw Venn diagram software. Three datasets (GSE33113, GSE23878, and GSE41328) were used and indicated by different colors. The overlapping area indicated common DEGs. **(A)** Twenty-two DEGs were up-regulated in all three datasets (logFC> 0). **(B)** Eighty-three DEGs were down-regulated in three datasets (logFC <0).

There were 127 CRC tissues and 32 normal colorectal tissue samples used in this study. We obtained 990, 720, and 280 DEGs from GSE33113, GSE23878, and GSE41328, respectively, using the GEO2R online tool. After integrated analysis, a total of 105 DEGs were identified as common to the three databases. These included 22 up-regulated DEGs (logFC> 0) and 83 down-regulated DEGs (logFC< 0) in the CRC tissues when compared with the normal colorectal tissues (Table 1 and Figure 1).

**TABLE 1:** The 105 common DEGs were identified from the three gene expression profile datasets.

DEGs	Gene names
Upregulated	ADAM12, NFE2L3, CEMIP, GDF15, CTHRC1, TRIB3, TACSTD2, FOXQ1, MMP3, TESC, CXCL5, ASCL2, BGN, INHBA, AJUBA, MMP1, WISP1, CXCL8, CRNDE, CLDN1, MMP11, SLC O 4A1
Downregulated	LGALS2, NR3C2, SPIB, HSD17B2, ABCG2, HMGCS2, ZG16, GUCA2B, UGT2B17, CHP2, SCARA5, CLCA4, DHRS11, AKR1B10, TUBAL3, ARL14, CA4, TRPM6, NXPE4, IGH, PTGDR, PYY, UGT2B15, SCIN, SLC26A3, B3GALT5, TSPAN7, HHLA2, CA2, DPP10-AS1, FCGBP, CHGA, SLC26A2, PKIB, ANPEP, CEACAM7, PADI2, C10orf99, ADTRP, NR1H4, KLF4, ISX, ABCA8, MUC2, BEST2, SLC51B, ADH1B, EDN3, AQP8, GCG, LYPD8, CD177, GBA3, MS4A12, PCK1, VSIG2, ADH1C, TMEM72, HEPACAM2, UGT2A3, GCNT2, LRRC19, SST, SCNN1B, NXPE1, C2orf88, HPGD, LAMA1, CWH43, BEST4, CA1, STMN2, LOC100506558///MATN2, MUC4, SLC4A4, MOGAT2, CA12, SI, SLC51A, GUCA2A,UGT1A3///UGT1A1///UGT1A4///UGT1A9///UGT1A5///UGT1A6///UG, T1A7///UGT1A8///, GT1A10, DHRS9, CA7

### GO and KEGG Pathway Enrichment Analyses of Common DEGs in CRC

All 22 up-regulated DEGs were analyzed using DAVID online resources. The GO analysis showed that the highest proportion of up-regulated DEGs were involved in 1) collagen catabolic process, cell-cell signaling, extracellular matrix disassembly, negative regulation of protein kinase activity, and positive regulation of protein oligomerization, found in the BP category; 2) proteinaceous extracellular matrix, extracellular region, extracellular space and extracellular matrix, found in the CC category; 3) metalloendopeptidase activity, protein kinase inhibitor activity, serine-type endopeptidase activity and transforming growth factor-beta receptor binding, found in the MF category (Table 2).

**TABLE 2:** Gene ontology analysis results of up-regulated DEGs in CRC.

Category	Term	Count	P-Value	FDR
GOTERM_BP_DIRECT	GO:0030574∼collagen catabolic process	3	0.002599	3.222424
GOTERM_BP_DIRECT	GO:0007267∼cell-cell signaling	4	0.003223	3.981161
GOTERM_BP_DIRECT	GO:0022617∼extracellular matrix disassembly	3	0.003643	4.489588
GOTERM_BP_DIRECT	GO:0006469∼negative regulation of protein kinase activity	3	0.006101	7.412011
GOTERM_BP_DIRECT	GO:0032461∼positive regulation of protein oligomerization	2	0.016552	18.94565
GOTERM_CC_DIRECT	GO:0005578∼proteinaceous extracellular matrix	6	8.57E-06	0.007978
GOTERM_CC_DIRECT	GO:0005576∼extracellular region	10	2.19E-05	0.020359
GOTERM_CC_DIRECT	GO:0005615∼extracellular space	7	0.002534	2.333393
GOTERM_CC_DIRECT	GO:0031012∼extracellular matrix	3	0.041212	32.40586
GOTERM_MF_DIRECT	GO:0004222∼metalloendopeptidase activity	4	3.06E-04	0.30983
GOTERM_MF_DIRECT	GO:0004860∼protein kinase inhibitor activity	3	0.001707	1.714626
GOTERM_MF_DIRECT	GO:0004252∼serine-type endopeptidase activity	3	0.036114	31.09485
GOTERM_MF_DIRECT	GO:0005160∼transforming growth factor-beta receptor binding	2	0.048628	39.63498

All 83 down-regulated DEGs were also analyzed using the DAVID online database. The GO analysis indicated that the highest proportion of down-regulated DEGs are enriched in 1) bicarbonate transport, one-carbon metabolic process, regulation of intracellular pH, chloride transmembrane transport, and cellular glucuronidation, found in the BP category; 2) an integral component of the plasma membrane, apical plasma membrane, anchored component of the membrane and integral component of membrane, found in the CC category; 3) carbonate dehydratase activity, chloride channel activity, hormone activity, transporter activity and glucuronosyltransferase activity, found in the MF category (Table 3).

**TABLE 3:** Gene ontology analysis results of down-regulated DEGs in CRC.

Category	Term	Count	p-Value	FDR
GOTERM_BP_DIRECT	GO:0015701∼bicarbonate transport	8	4.43E-10	6.17E-07
GOTERM_BP_DIRECT	GO:0006730∼one-carbon metabolic process	5	6.23E-06	0.008676
GOTERM_BP_DIRECT	GO:0051453∼regulation of intracellular pH	4	4.12E-04	0.572874
GOTERM_BP_DIRECT	GO:1902476∼chloride transmembrane transport	5	5.48E-04	0.760588
GOTERM_BP_DIRECT	GO:0052695∼cellular glucuronidation	3	0.001869	2.574031
GOTERM_CC_DIRECT	GO:0005887∼integral component of plasma membrane	14	0.006041	6.366143
GOTERM_CC_DIRECT	GO:0016324∼apical plasma membrane	6	0.007776	8.124723
GOTERM_CC_DIRECT	GO:0031225∼anchored component of membrane	4	0.012178	12.4538
GOTERM_CC_DIRECT	GO:0016021∼integral component of membrane	32	0.015945	16.01086
GOTERM_MF_DIRECT	GO:0004089∼carbonate dehydratase activity	5	2.34E-07	2.78E-04
GOTERM_MF_DIRECT	GO:0005254∼chloride channel activity	5	6.54E-05	0.077743
GOTERM_MF_DIRECT	GO:0005179∼hormone activity	5	5.59E-04	0.662534
GOTERM_MF_DIRECT	GO:0005215∼transporter activity	6	0.001321	1.559094
GOTERM_MF_DIRECT	GO:0015020∼glucuronosyltransferase activity	3	0.006051	6.960171

We also performed a KEGG pathway analysis using the DAVID online database. This indicated that up-regulated DEGs were particularly enriched in rheumatoid arthritis, cytokine-cytokine receptor interaction, and bladder cancer pathways, whilst the nitrogen metabolism, bile secretion, retinol metabolism, proximal tubule bicarbonate reclamation, and drug metabolism pathways were identified as the most represented pathways for the down-regulated DEGs (Table 4, *P* < 0.05).

**TABLE 4:** The KEGG pathways involved with the common DEGs in CRC.

Pathway id	Name	Count	P-Value	Genes
Up-regulated				
hsa05323	Rheumatoid arthritis	4	6.82E-05	CXCL5, CXCL8, MMP3, MMP1
hsa04060	Cytokine-cytokine receptor interaction	3	0.023207	INHBA, CXCL5, CXCL8
hsa05219	Bladder cancer	2	0.041	CXCL8, MMP1
Down-regulated				
hsa00910	Nitrogen metabolism	5	2.97E-06	CA12, CA7, CA4, CA2, CA1
hsa04976	Bile secretion	7	3.72E-06	AQP8, SLC51B, CA2, SLC51A, SLC4A4, NR1H4, ABCG2
hsa00830	Retinol metabolism	6	4.36E-05	UGT2B17, ADH1C, DHRS9, ADH1B, UGT2A3, UGT2B15
hsa04964	Proximal tubule bicarbonate reclamation	4	3.69E-04	CA4, CA2, SLC4A4, PCK1
hsa00982	Drug metabolism - cytochrome P450	5	8.06E-04	UGT2B17, ADH1C, ADH1B, UGT2A3, UGT2B15

### PPI Network Complex and Hub Gene Analysis

**FIGURE 2:**
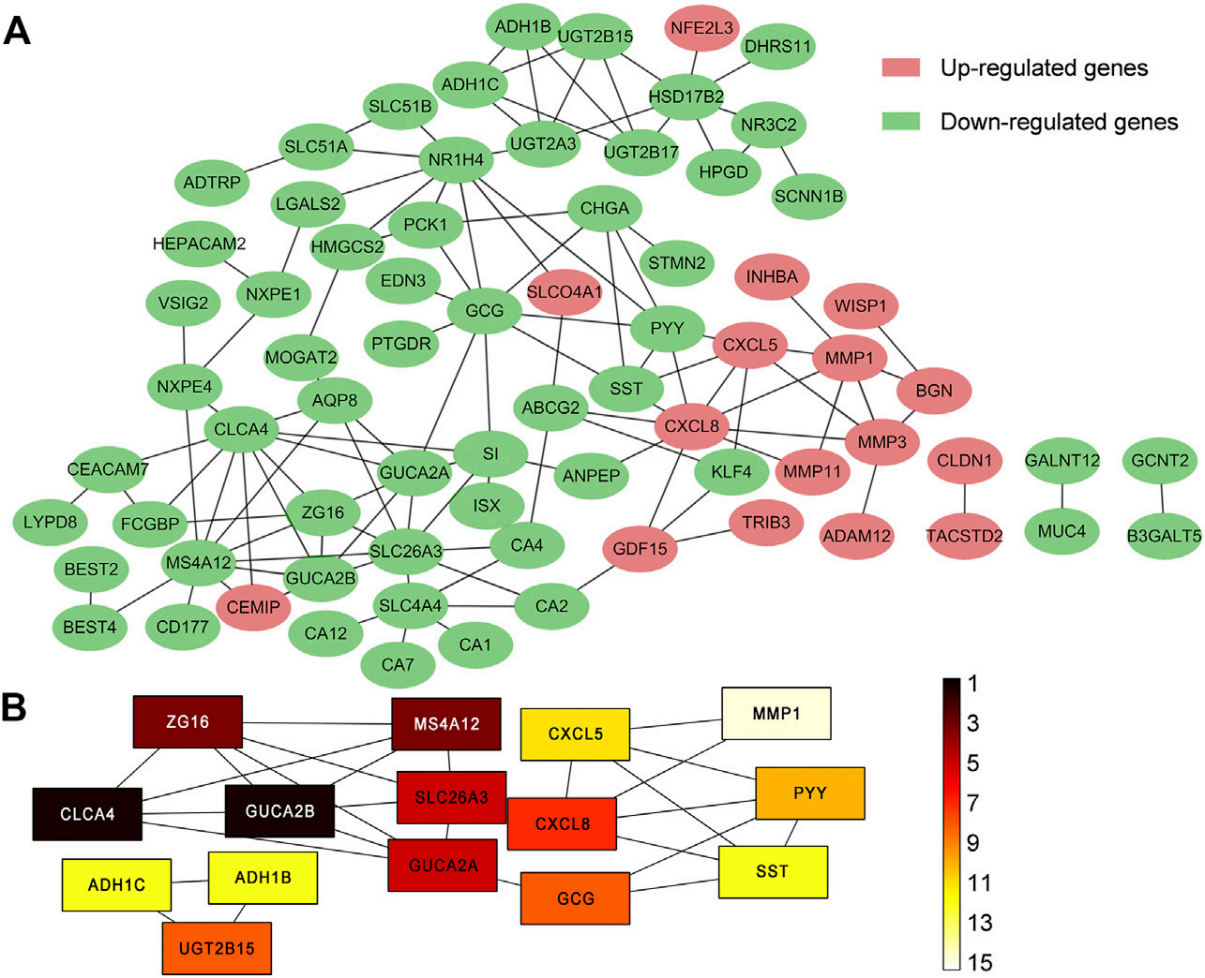
PPI network of 105 common DEGs built by STRING and Cytoscape module analysis. **(A)** 73 out of the 105 DEGs were contained in the PPI network complex. The 73 nodes and 122 edges represent the interaction of proteins. Red circles indicate the up-regulated DEGs and green circles indicate the down-regulated DEGs; **(B)** The rankings of the top 15 hub genes identified by the Cytoscape plugin cytoHubba MCC. 9 different colors from black to white was chosen to show the rank of the hub genes.

Among the 105 common DEGs, 73 were used to construct a PPI complex of 73 nodes and 122 edges using STRING (http://string-db.org) and Cytoscape software. The 73 genes included 16 up-regulated and 57 down-regulated genes, whilst the remaining 32 genes were not found in a PPI network complex (Figure 2A). The top 15 ranking hub genes were selected (ADH1B, ADH1C, CLCA4, CLCX5, CXCL8, GCG, GUCA2A, GUCA2B, MS4A12, MMP1, PYY, SLC26A3, SST, UGT2B15 and ZG16) by the MCC algorithm of the cytoHubba application for further research (Table 5; Figure 2B).

**TABLE 5:** The hub genes with top15 ranking of the PPI network.

Hub genes	Protein name	Rank
GUCA2B	Guanylate cyclase activator 2B	1
CLCA4	Chloride channel accessory 4	1
ZG16	Zymogen granule protein 16	3
MS4A12	Membrane spanning 4-domains A12	3
GUCA2A	Guanylate cyclase activator 2A	5
SLC26A3	Solute carrier family 26 member 3	5
CXCL8	C-X-C motif chemokine ligand 8	7
GCG	Glucagon	8
UGT2B15	UDP glucuronosyltransferase family 2 member B15	8
PYY	Peptide YY	10
CXCL5	C-X-C motif chemokine ligand 5	11
ADH1C	Alcohol dehydrogenase 1C (class I), gamma polypeptide	12
ADH1B	Alcohol dehydrogenase 1B (class I), beta polypeptide	12
SST	Somatostatin	12
MMP1	Matrix metallopeptidase 1	15

### Validation of Hub Genes by GEPIA

**FIGURE 3:**
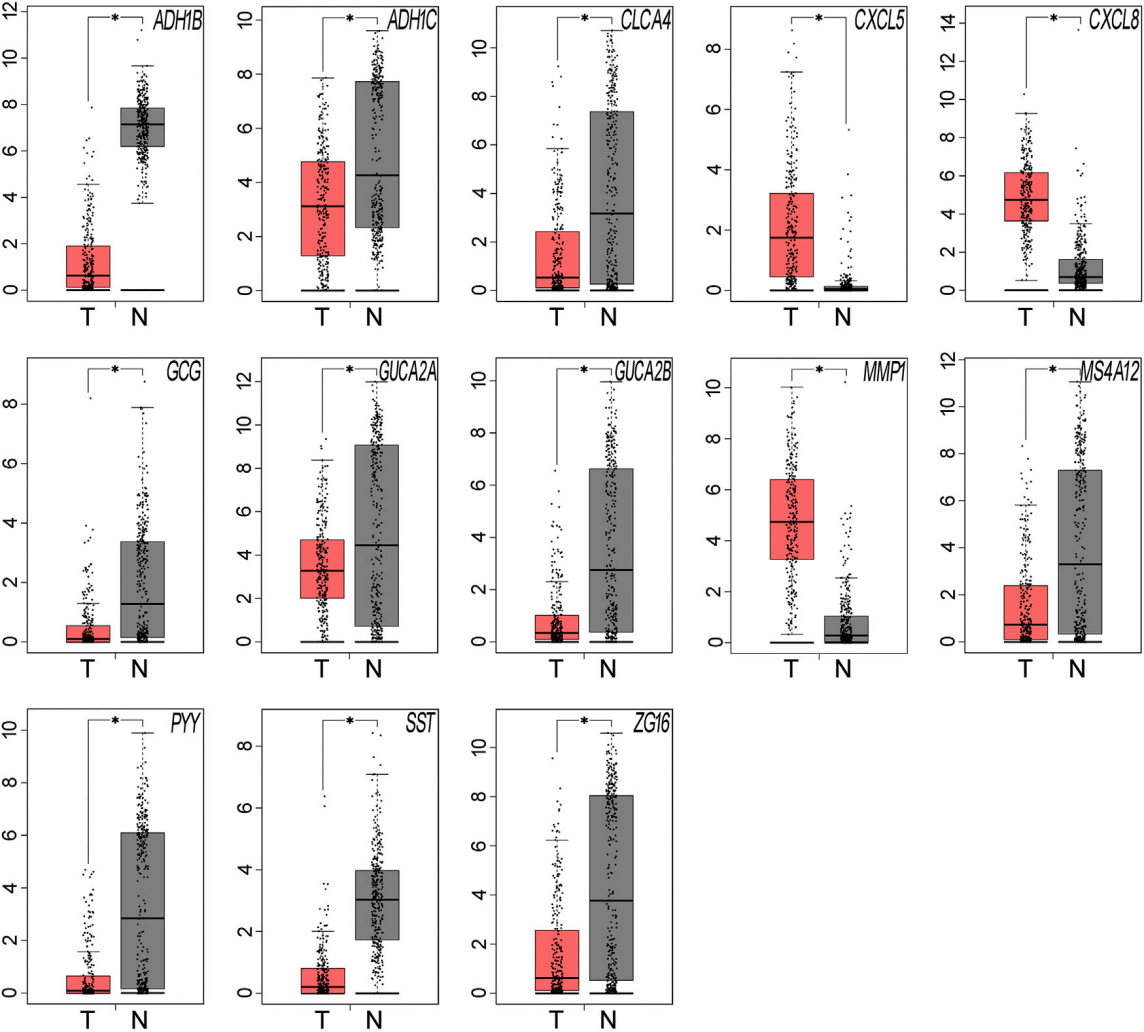
The expression levels of the hub genes were validated using the GEPIA website. Thirteen of the 15 hub genes showed significant differential expression in CRC samples, when compared with the normal samples (*P < 0.05). Red represents tumor tissues and grey represents normal tissues.

We used GEPIA to compare the expression levels of the 15 hub genes between samples from CRC patients and normal individuals. The results showed that 13 out of the 15 hub genes had significantly different expression levels in CRC samples when compared to normal colorectal mucosa tissues (*P* < 0.05, Table 6 and Figure 3). SLC26A3 and UGT2B15 did not show differential expression (Table 6).

**TABLE 6:** Validation of 13 hub genes using GEPIA.

Category	Genes
Genes with differential expression between the CRC and normal tissues (*p* < 0.05)	ADH1B, ADH1C, CLCA4, CXCL5, CXCL8, GCG, GUCA2A, GUCA2B, MMP1, MS4A12, PYY, SST, ZG16
Genes without differential expression between the CRC and normal tissues (*p* > 0.05)	SLC26A3, UGT2B15

### Prognostic Analysis of the Validated Hub Genes by UALCAN and OncoLnc

**FIGURE 4:**
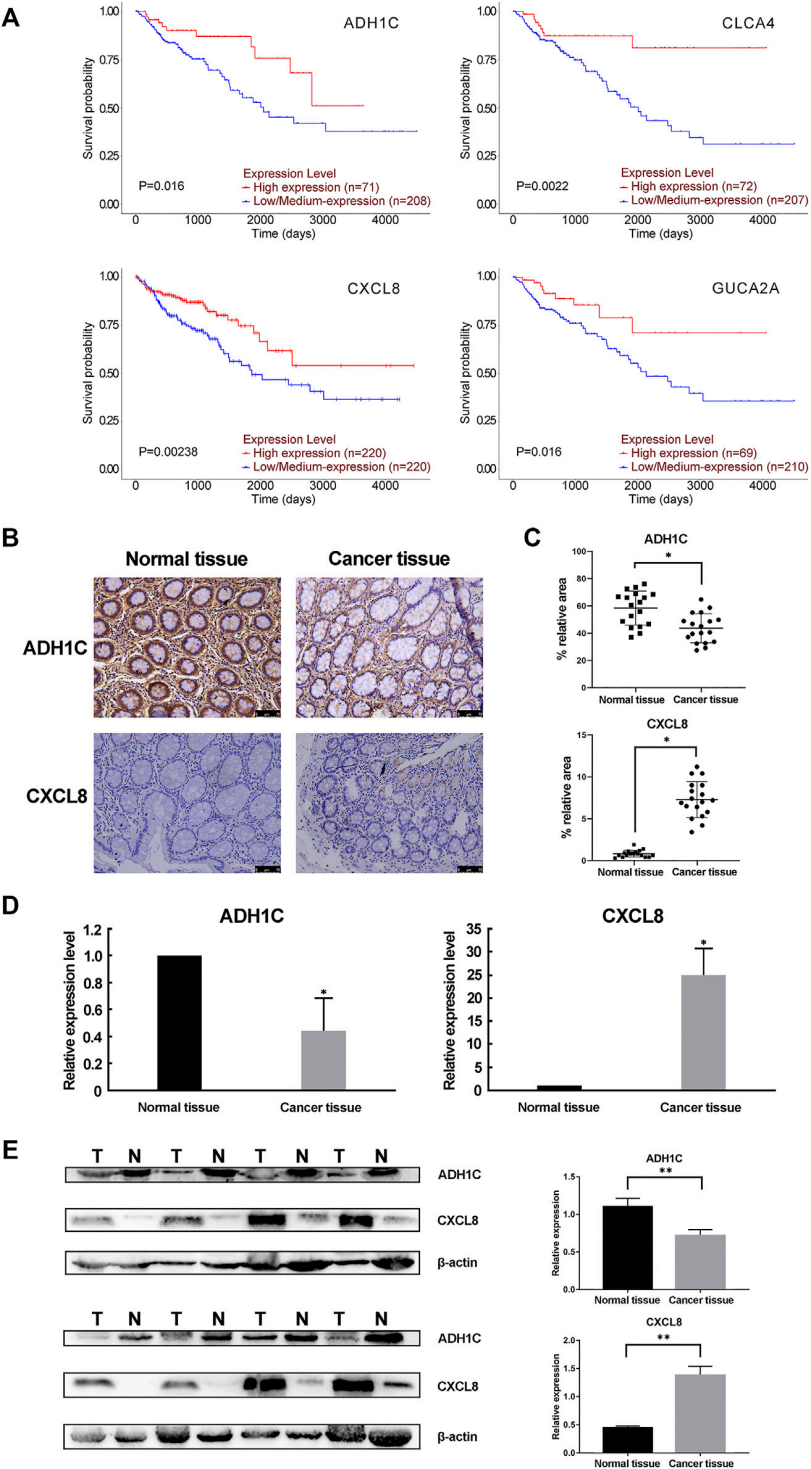
The prognostic analysis and the protein level of the hub genes. **(A)** UALCAN and OncoLnc online resources were applied to obtain the prognostic results of the 13 hub genes. Only four of the 13 hub genes had significantly lower overall survival rates (*P* < 0.05). **(B,C)** Immunohistochemistry (IHC) analysis of CRC tissue and adjacent normal tissue. **(B)** showed the representative DAB IHC staining with ADH1C, CXCL8 antibody, and cell nuclei stained by hematoxylin of cancer and normal tissue pathology slides. **(C)** showed the quantitative analysis results by ImageJ and IHC Toolbox plugin. The magnification of the field is ×100, and the scale bar is 75 μm. **(D)** Real-time PCR results confirmed the dysregulation of the mRNA expression of ADH1C and CXCL8. **(E)** The protein level of ADH1C and CXCL8 were detected by Western blot. T, tumor tissue; N, normal tissue. *n* = 8. **P < 0.01 or *P < 0.05 by paired t-test.

We performed a prognostic analysis of the 13 validated hub genes using the UALCAN (http://ualcan.path.uab.edu/) online resource database. The results showed that low expression of ADH1C, CLCA4, and GUCA2A was associated with significantly lower survival rates. There was no available data for CXCL8 and the other nine genes showed no significant changes (*P* < 0.05, Table 7 and Figure 4A). However, down-regulated CXCL8 was also significantly related to a lower survival rate (*P* < 0.05, Figure 4A), as shown by an analysis on the OncoLnc (http://www.oncolnc.org/) open online database. Furthermore, we collected CRC tissues and matched normal colorectal tissues from the Affiliated Hospital of Hebei University. The immunohistochemical analyses, real-time PCR and western blot confirmed that both the mRNA and protein level of ADH1C was down-regulated, well CXCL8 was up-regulated in the CRC tissues compared with normal colorectal tissues (*P* < 0.05, Figures 4B–E).

**TABLE 7:** The prognostic analysis results of the 13 hub validated genes.

Category	Genes
Genes with significantly worse survival (*P* < 0.05)	ADH1C, CLCA4, CXCL8, GUCA2A
Genes without significantly worse survival (*P* > 0.05)	ADH1B, GCG, GUCA2B, MMP1, MS4A12, PYY, SST, ZG16, CXCL5

## Discussion

Numerous studies have focused on the pathology and mechanism of CRC, but the exact mechanism remains largely unknown. To investigate the genes related to pathogenesis and to address the urgent need for early-stage diagnostic biomarkers for CRC, we integrated three gene profile datasets: GSE33113, GSE23878, and GSE41328, from GEO. 22 up-regulated and 83 down-regulated genes were identified when CRC samples were compared with colorectal tissues.

We performed GO and KEGG pathway enrichment analyses using DAVID online resources. Most up-regulated genes had functions that are integral to the extracellular region and extracellular space, which suggested that the formation of CRC has a close relationship with the tumor microenvironment. The largest proportion of down-regulated genes was mainly involved in the integral component of the membrane, an integral component of the plasma membrane, and bicarbonate transport. This is consistent with the fact that the integral cell membrane is essential for the avoidance of pathogens and to maintain the acid-base balance (Than et al., 2016; Westman et al., 2019). In the KEGG pathway analysis, up-regulated genes were particularly enriched in rheumatoid arthritis and the cytokine-cytokine receptor interaction. This is consistent with the GO analysis result of the up-regulated genes because the extracellular matrix and cytokine signal transduction play an important role in CRC invasion and metastasis (Cabrero-de Las Heras and Martinez-Balibrea, 2018; Yuzhalin et al., 2018). The highest proportion of down-regulated genes was significantly associated with nitrogen metabolism and bile secretion. Reactive nitrogen may function as a pro-inflammatory factor that promotes CRC (Janakiram and Rao, 2014). We also noted that many studies have shown that the gut microbiota that participates in the bile acid metabolism takes part in the formation of CRC (Feng et al., 2015; Wong et al., 2017), which is consistent with bile acid being associated with the occurrence of CRC (Liu et al., 2019).

We found that 13 out of the 15 hub genes showed differential expression in CRC samples when compared to normal samples. This was validated using GEPIA (*P* < 0.05). However, only four (ADH1C, CLCA4, CXCL8, GUCA2A) out of the 13 genes were significantly associated with a lower survival rate. The lower protein level of ADH1C and higher protein level of CXCL8 were further verified in our CRC and matched normal colorectal samples.

ADH1C, also known as ADH3, is an alcohol dehydrogenase that can catalyze ethanol oxidation to metabolite acetaldehyde (Shen et al., 2019). According to THE HUMAN PROTEIN ATLAS database, ADH1C locates mainly to the cytosol and plasma membrane, which is consistent with our IHC result. Genetic polymorphism of the ADH1C gene (ADH1C*1 allele) is associated with various human cancers, such as gastric cancer (Hidaka et al., 2015), oral cancer (Anantharaman et al., 2014) and CRC (Offermans et al., 2018), for it encodes an alcohol dehydrogenase producing 2.5 times more acetaldehyde (Seitz and Stickel, 2010). Chiang et al. showed that ADH1C was the mainly expressed isozyme in colorectal tissues (Chiang et al., 2012). Mashkova et al. observed that lower mRNA level of ADH1C in the CRC tissues compared with the normal or only hyperplasia colorectal tissues (Kropotova et al., 2014). In addition, the latest study showed that ADH1C is down-regulated in familial adenomatous polyposis case adenomas (Thiruvengadam et al., 2019), but up-regulated in patients with ulcerative colitis (Low et al., 2019). Kumamoto et al. (2019) revealed that ADH1C could predict the recurrence of stage III CRC in patients who accept chemotherapy treatment. Our study is the first to show a correlation between the lower expression level of ADH1C and worse prognostic of CRC patients. What’s more, we conducted immunohistochemical analyses and western blot to testify that ADH1C was down-regulated in CRC tissues in comparison with the normal tissues consisting with our bioinformatics analysis. However, there are few studies supporting our conclusion, further experiments are needed to verify our results.

Chloride channel accessory 4 (CLCA4) is well known as a tumor inhibitor and has been shown to suppress the development of various malignant tumors. In bladder cancer and hepatocellular carcinoma, tumor cell proliferation and migration are inhibited by CLCA4 through the PI3K/AKT signalling pathway (Hou et al., 2017; Liu et al., 2018). Low expression of CLCA4 has been reported in CRC patients (Zhao et al., 2019). Chen et al. (2019) reported that the epithelial-mesenchymal transition can also be suppressed by CLCA4 *via* the PI3K/AKT pathway in CRC, and also indicated that low CLCA4 is correlated with poor survival of CRC patients. This is consistent with our results.

The C-X-C motif chemokine ligand 8 (CXCL8) gene belongs to the CXC chemokine family whose main function is to recruit and activate neutrophils and granulocytes to sites of inflammation (Waugh and Wilson, 2008). Several previous studies have shown that CXCL8 which is a secreted protein functions with its receptors, CXCR1 and CXCR2, to promote the progression of some cancers, such as breast cancer (Yi et al., 2019), prostate cancer (Baci et al., 2019), lung cancer (He et al., 2019), and CRC (Liu et al., 2016). The CXCL8 gene is up-regulated in CRC tissue and correlated with the development of CRC, which is physiologically hard to detect (Rubie et al., 2007). Xia et al. (2015) proved that high levels of expression of CXCL8 are significantly associated with poor overall survival, tumor stage, lymphatic and liver metastasis. This suggests that CXCL8 could be a potential indicator for both detection and prognosis by meta-analysis. Fisher et al. (2019) demonstrated that blocking the CXCL8-CXCR1 pathway can inhibit the tumorigenicity that originates in the CRC stem cells. However, we did observe elevated CXCL8 levels in CRC patients in our study, the bioinformatics analysis indicated that a high level of CXCL8 correlated with poor prognosis at the beginning and in related with longer survival time as the time lasted in the opposite. Therefore, more studies are needed to investigate the exact relationship between the expression of CXCL8 and the CRC.

Guanylate cyclase activator 2A (GUCA2A) is a peptide hormone that is secreted by gut epithelial cells to regulate the function of guanylate cyclase 2C (GUCY2C) signalling in the autocrine and paracrine systems (Pattison et al., 2016). Silencing of the GUCY2C receptor induces genomic instability, hyperproliferation, and transformation of tumor cells (Li et al., 2007; Lin et al., 2016). Bashir et al. (2019) showed that a low level of GUCA2A silences the tumor inhibitory receptor, GUCY2C, in pathophysiological conditions, and leads to microsatellite instability in tumors. Loss of GUCA2A has been observed in CRC and inflammatory bowel disease, in which it may be associated with the disruption of intestinal homeostasis (Brenna et al., 2015; Zhang et al., 2019). Zhang et al. (2019) used analysis of the TCGA database to show that GUCA2A is associated with poor overall survival, which is consistent with our results.

## Conclusion

In conclusion, our bioinformatics study identified significantly enriched KEGG pathways and four common DEGs (ADH1C, CLCA4, CXCL8, and GUCA2A) that correlate with poor overall survival of CRC patients. Our GO and KEGG pathways analyses indicated that the tumor microenvironment and gut microbiota are involved in the progression of CRC. We confirmed the decreased level of ADH1C in our CRC tissues from our patients and we are also the first to reveal the relationship between ADH1C and the lower overall survival rate of CRC patients. This study provides information on the pathogenesis of CRC and indicates that ADH1C may be a candidate prognostic molecular. More empirical and clinical verifications are needed to add to our analyses and further studies on the mechanism of the disease are also necessary. In short, our results have identified potential prognostic markers for CRC and are also shed light on the mechanism of CRC.

## Data Availability

Publicly available datasets were analyzed in this study. This data can be found here: https://www.ncbi.nlm.nih.gov/gds/?term=GSE33113; https://www.ncbi.nlm.nih.gov/geo/query/acc.cgi?acc=GSE23878; https://www.ncbi.nlm.nih.gov/geo/query/acc.cgi?acc=GSE41328.
